# Simultaneous measurements of kinematics and fMRI: compatibility assessment and case report on recovery evaluation of one stroke patient

**DOI:** 10.1186/1743-0003-7-49

**Published:** 2010-09-23

**Authors:** Claudia Casellato, Simona Ferrante, Marta Gandolla, Nicola Volonterio, Giancarlo Ferrigno, Giuseppe Baselli, Tiziano Frattini, Alberto Martegani, Franco Molteni, Alessandra Pedrocchi

**Affiliations:** 1 Politecnico di Milano, Bioengineering Dept., NearLab, piazza L. Da Vinci 32, 20133, Milano, Italy; 2 Politecnico di Milano, Bioengineering Dept., piazza L. Da Vinci 32, 20133, Milano, Italy; 3 Valduce Hospital, Unità operativa complessa di Radiologia, via D. Alighieri 11, 22100, Como, Italy; 4 Valduce Hospital, Villa Beretta, Unità operativa complessa di medicina riabilitativa, via N. Sauro 17, 23845, Costamasnaga (LC), Italy

## Abstract

**Background:**

Correlating the features of the actual executed movement with the associated cortical activations can enhance the reliability of the functional Magnetic Resonance Imaging (fMRI) data interpretation. This is crucial for longitudinal evaluation of motor recovery in neurological patients and for investigating detailed mutual interactions between activation maps and movement parameters.

Therefore, we have explored a new set-up combining fMRI with an optoelectronic motion capture system, which provides a multi-parameter quantification of the performed motor task.

**Methods:**

The cameras of the motion system were mounted inside the MR room and passive markers were placed on the subject skin, without any risk or encumbrance. The versatile set-up allows 3-dimensional multi-segment acquisitions including recording of possible mirror movements, and it guarantees a high inter-sessions repeatability.

We demonstrated the integrated set-up reliability through compatibility tests. Then, an fMRI block-design protocol combined with kinematic recordings was tested on a healthy volunteer performing finger tapping and ankle dorsal- plantar-flexion. A preliminary assessment of clinical applicability and perspectives was carried out by pre- and post rehabilitation acquisitions on a hemiparetic patient performing ankle dorsal- plantar-flexion. For all sessions, the proposed method integrating kinematic data into the model design was compared with the standard analysis.

**Results:**

Phantom acquisitions demonstrated the not-compromised image quality. Healthy subject sessions showed the protocols feasibility and the model reliability with the kinematic regressor. The patient results showed that brain activation maps were more consistent when the images analysis included in the regression model, besides the stimuli, the kinematic regressor quantifying the actual executed movement (movement timing and amplitude), proving a significant model improvement. Moreover, concerning motor recovery evaluation, after one rehabilitation month, a greater cortical area was activated during exercise, in contrast to the usual focalization associated with functional recovery. Indeed, the availability of kinematics data allows to correlate this wider area with a higher frequency and a larger amplitude of movement.

**Conclusions:**

The kinematic acquisitions resulted to be reliable and versatile to enrich the fMRI images information and therefore the evaluation of motor recovery in neurological patients where large differences between required and performed motion can be expected.

## Background

Functional magnetic resonance imaging (fMRI) is one of the main tools to investigate brain functional responses and follow-up their evolution. Its non-invasiveness, flexibility, spatial resolution, and reference to MRI anatomical images allows functional standard localizations. However, the analysis of fMRI performed during motor tasks in neurological patients affected by movement impairments (e.g. hemiparesis) requires an adequate monitoring of the actual executed movement performance and timing. Indeed, the required task could be incorrectly carried out and involuntary movements could occur. Moreover, longitudinal studies require repeatability of motor tasks performed in different sessions, in order to not confuse changes in the execution of the movements with evolutions in the brain functional response. Furthermore, mirror movements, i.e., unintentional and simultaneous replication on the healthy side of the intended movements performed by the paretic side, are quite common [1] and can affect the interpretation of obtained images.

Several studies focusing on motor protocols under fMRI examination applied different methods to acquire movement performance outcomes. Many fMRI studies used visual inspection [2,3], sometimes coupled to palpation [4], to evaluate subject's compliance to the requested task; obviously these methods are only qualitative. Other studies used electrogoniometers [5,6] or ShapeTape™ (Measurand Inc., Fredericton, NB) [7] to measure the angle at the ankle. Both these devices measure only in one plane, and are cumbersome and not suitable for multi-joint acquisitions. Horenstein et al. [8] recorded finger tapping performance with a MR compatible glove (Fifth Dimension Technologies, Irvine, CA); wearing a glove could, however, generate discomfort in subjects and limit their freedom in the execution of movements. In some studies forces produced by the subject were recorded using a pressure transducer built in a hydraulic environment [9,10] or a load cell [11]. In case of force measure no free moving tasks can be executed.

Electromyography (EMG) is a very complete method to monitor the neuro-motor output [12] because even an isometric contraction and a low contraction unable to produce a visible movement can be detected. Indeed, in most of the latest fMRI studies, EMG has been employed [9,10].

Until a few years ago, it was hard to get reliable EMG signals: indeed, the EMG recordings under the high fMRI fields are corrupted by induction artifacts, highly correlated to the movement and thus, hardly separable from the addressed EMG. Initially, EMG was analyzed only during a short inter-scan interval and used as a time trigger, avoiding any quantitative measurement. Nowadays [12-14] new artifacts correction techniques were validated, leading to achieve a reliable EMG signal recorded even during scanning periods [15]. Recently, Van Duinen and colleagues [16] showed activity in the motor areas strongly correlated with muscle activity during contractions at different force levels. Nonetheless EMG could have potential risks for the subjects due to the contact of skin with metallic parts inside time-varying magnetic field and the MR compatibility leads to a significant rising of costs. However, inter-session repeatability of EMG signal recorded in MRI environment is very limited, mainly because it strongly depends on electrodes placement.

Exploring a different approach to the same goal, this study intended to develop a new set-up which combines a fMRI system with an optical motion capture system. The motion capture system records 3-D trajectories of passive markers with high accuracy [17]. The proposed integrated system has different advantages with respect to the commonly used technologies. First, it allows to calibrate wide working volume so to acquire multi-segment tasks. Second, the only direct contact elements with the patient are small, light and plastic markers, which do not limit spontaneous movement execution and do not carry any potential risk for the subject. Third, the recorded trajectories of the markers are very reliable and highly accurate and well established data processing permit to calculate angular ranges of motions, velocities and accelerations in 3-D of all the segments, enriching the fMRI activation maps with a complete description of the kinematics of the motor output. Fourth, markers placement is very reliable assuring the intersession repeatability.

The present work aims at proving the mutual compatibility of using a motion capture system inside the MRI bore, by phantom tests and healthy subject acquisitions before and after motion capture insertion. Secondly, it aims at proposing a method to utilize the recorded kinematics parameters into the fMRI model design, adding movement output as regressor, and to demonstrate the possible positive impact, especially in a neurological (partly collaborative) subject at different stages of rehabilitation.

## Methods

### Participants

Two acquisition sessions were performed on a healthy subject (24 years old, male, right-handed), both to assess compatibility between the motion capture and fMRI and to evaluate the feasibility of different motor tasks as clinical protocols.

One hemiparetic subject was recruited to validate the clinical usefulness of the setup. The patient (61 years old, female, right-handed) suffered from an ischemic stroke 4 weeks before the hospitalization in the rehabilitation center. Lesion was located on the right hemisphere and covered the insula and temporopolar cortex. She was not claustrophobic and she had no implanted devices incompatible with MR.

fMRI acquisitions were performed at the hospitalization and after one month of rehabilitation therapy. She underwent standard rehabilitation treatment (passive and active movements) and 20 functional electrical stimulation cycling sessions [18].

Here we report some clinical scores, representative of her motor impairment.

• At hospitalization (pre). Motricity Index on the lower limbs = 26; quadriceps forces produced during a maximal voluntary isometric contraction: for right side (healthy) = 112 N, for left side (paretic) = 13N.

• After one month (post). Motricity Index on the lower limbs = 45; quadriceps forces produced during a maximal voluntary isometric contraction: right = 140 N, left = 52 N.

This study was undertaken with the understanding and written consent of each subject, with the approval of the Ethical Board of Villa Beretta Rehabilitation Centre.

### fMRI

MRI was performed on a GE Cv/I™ 1.5 T scanner. Subjects anatomy was acquired with a 3D spoiled gradient echo sequence T1-weighted; echo time (TE) = 6.9 ms; automatic repetition time (TR) = 15.9 ms; flip angle = 15°; matrix 256×256; field of view (FOV) = 26 cm; voxel size = 1×1×0.8 mm.

For functional imaging sessions a gradient EPI sequence T2-weighted was used; TE = 50 ms; TR = 3 s; flip angle = 90°; matrix 128×128; FOV = 24 cm; voxel size = 1.8×1.8×4 mm.

Each functional acquisition included 100 volumes of 22 images, for a total of 2200 scans.

### Motion Capture System

A motion capture system, Smart μg™ (BTS, Italy), was used to measure kinematics. Cameras have a CCD detector sensible to infrared and a LED enlighter emitting at 850 nm; the working frequency was set to 60 Hz. The system works with passive plastic retroreflective markers, which reflect the near-infrared light allowing the cameras to detect their 2D projection on the sensor planes. From the calibration parameters of each camera and the marker 2D coordinates coming from at least two cameras sensors at the same time instant, the system algorithm is able to provide the absolute 3D position of each marker, by collinearity equations [17]. Then, the tracking procedure is performed by the operator, using a system-specific software (SmartTracker^®^), in order to associate the 3D reconstructed data with the markers model, along all acquired frames.

**Figure 1:**
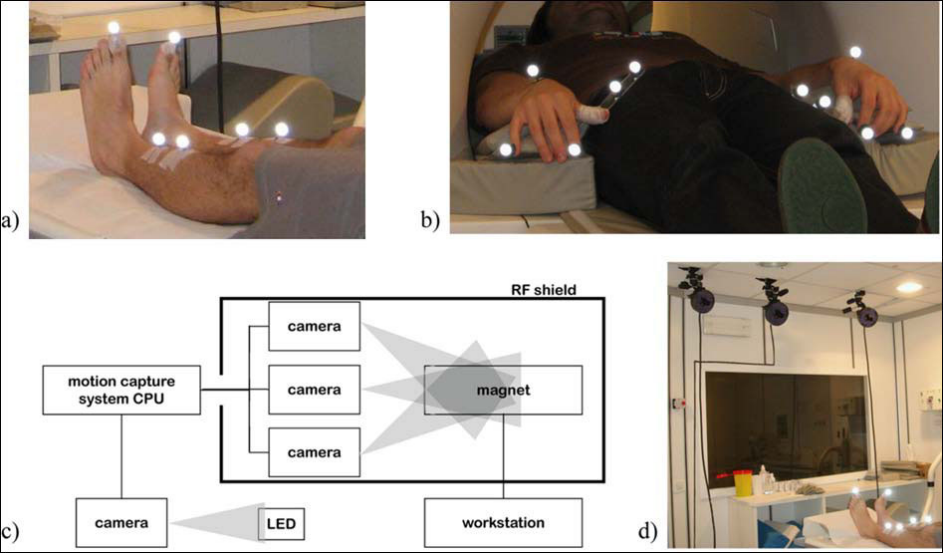
**Set-up**. a) position of the markers for ankle dorsal- plantar-flexion acquisitions; b) position of the markers for finger tapping acquisitions; c-d) a scheme and a photo of the integrated experimental setup.

In the present set-up three cameras were bounded (with SuperClamp 035™ and 804RC2™ heads Manfrotto, Italy) to the MR room ceiling, inside the Radio-Frequency (RF) shield, with one camera centered above the axis of the bore and the other two 1.0 m apart on each side, at the maximum possible distance from the bore (about 3 m). The working volume was about 1×1×1 m, the accuracy reconstruction was less than 1 mm. A fourth camera, outside the MRI room, was used to capture an active infrared LED, which was switched on simultaneously with the fMRI scanning start, in order to synchronize the fMRI protocol and the kinematics acquisition. Also the CPU was placed outside the shielded room, next to the radiologist desk. Cables connecting the CPU and the cameras located inside the MR room passed the RF shield across a waveguide (Fig. 1, panels c and d). The motion analyzer was calibrated with the shielded door opened; after calibration the door was closed and the fourth camera, used only for synchronization and not for movement reconstruction, was moved to capture the synchronizing LED.

Cameras, heads, clamps and cables are metallic; cameras and enlighters contain printed circuits which are sources of electromagnetic noise, as well as the cables. For this reason the integration of the two systems could introduce both RF noise and dishomogeneity in the main static magnetic field. As seen in literature [19], in order to limit the RF interference introduced into the MR images by electronic devices, aluminium foils, connected to MR room ground, were contiguously applied to the cables connecting cameras and CPU. Enlighters, as well, were partially covered with grounded aluminium foils. On the other hand, the optical components could be affected by the static magnetic field, provoking for instance a focalization degradation, and the electrical components could be compromised by the magnetic noise.

### Compatibility test

In order to evaluate the interference between the two systems, MR images of a phantom were acquired with and without the working motion capture system inside the MR room. A standard phantom with one-compartment of aqueous paramagnetic solutions was used. As for functional subjects acquisition, the gradient EPI sequence (with the parameters described above in *fMRI*) was performed. A 30 seconds session was acquired (TR = 3 s), thereby 10 volumes of 22 images each were obtained.

The Signal-to-Noise Ratio (SNR) was calculated on each slice for all volumes. We use the standard index for image quality [20], that is the ratio between the mean signal amplitude on a homogeneous area and the standard deviation of the background signal amplitude. Therefore, the ratio between the mean value of a small ROI placed in the most homogeneous area of phantom (around the barycentre) with high signal intensity and the mean of standard deviations for four ROIs placed outside the object in the image background was computed.

In order to get a change only depending from the presence of motion system, the acquisition parameters affecting the SNR were kept as in the reference acqusition: bandwidth, field of view, slice thickness, voxel volume, number of acquisitions (NEX) and number of scans.

The loss of SNR percentage was computed as following: ΔSNR = (SNR_ref _- SNR_system_)/SNR_ref _*100; where SNR_ref _corresponds to the reference condition and SNR_system _to the integrated set-up.

Moreover, we performed tests on kinematics data, in order to establish possible effects of magnetic fields on the recording accuracy of the motion capture system. A marker was repeatedly launched vertically during a phantom fMRI session. The equation of uniformly accelerated linear motion was applied on the descending tracks of the falling down marker: knowing, from recorded kinematic data, the displacement and duration, the mean value of acceleration was computed.

### Protocol procedures

Subjects were instructed to keep eyes closed to avoid activations of visual cortex. Head movements were minimized with rubber pads and straps. To ensure minimum transmission of movements to the head, across the spine, knees were bent and legs lied on a pillow. Participants wore earphone and microphone to communicate with the operator who gave them oral commands, triggering the task temporal sequence (start and stop of each 30 s block). The fMRI paradigm consisted of 5 resting epochs alternating with 5 activating ones. Each period lasted 30 s, thus the trial duration was 300 s.

Two different tasks, performed by the healthy subject, were used to evaluate the compatibility between the two systems and a preliminary clinical feasibility. The first task was the finger tapping. It was chosen because it is a well established task and it leads to the activation of well defined areas [9], easy to be localized. The healthy subject was asked to tap the thumb with the pulp of each finger in turn, and then start over again; no constraints were imposed on the frequency of execution. The second task was self-paced ankle dorsal- plantar-flexion. The subject performed the protocols alternatively with both sides.

For the hemiparetic patient only the ankle dorsal- plantar-flexion on both sides was chosen as clinical protocol for evaluation, since fine hand control was not completely recovered at the considered rehabilitation stage. In order to get confident with the required motor task, prior to each MRI acquisition, the patient underwent a training that replied, out of bore, the conditions of the examination. During this training, along with ankle angles of both limbs, superficial EMG signals from the soleus, the gastrocnemius lateralis and the tibialis anterior were acquired, in order to exclude mirror isometric contractions, which the kinematics system would not have detected.

### Kinematics acquisition and data analysis for finger tapping

Markers were placed on the top of the index and pinkie fingers and dorsally on the wrist (Fig. 1, panel b) of both hands. A plastic support with two markers identified each thumb; this solution was adopted to avoid uncorrected reconstructions, due to the compromising of markers visibility during the touching phases between fingers. Three fingers for each hand were considered sufficient for a validation acquisition on an healthy subject; indeed, desired movement parameters, as the frequency and the movement amplitude for each whole cycle, were computable. Since the subject was healthy, the accuracy of the task sequence (thumb sequential touches with index, middle, ring finger and pinkie) did not need to be verified on each of the four fingers.

The reconstructed trajectories were filtered with a fifth-order Butterworth low-pass filter (cutoff frequency = 5 Hz) and 3D displacements of index and pinkie fingers were analyzed. For each active period, considering all cycles, the mean Displacements of moving Index (ID) and of moving Pinkie (PD) were computed. The frequency (f) of movement (number of cycles for each 30 s block) was calculated; the same number of repetitions for the two analyzed fingers is a proof of correct task execution. The displacements for Index and Pinkie fingers not performing the task during activation epochs were estimated by Standard Deviations (ISD and PSD). To assess if the involuntary movements were mirror movements or not, the correlation coefficients (R indexes and R pinkies) between the two-hands corresponding finger displacements were computed. Movements of the hand which was required to stand still were considered significant when SDs > 0.5 cm, and were considered mirror movements when R > 0.5.

### Kinematics acquisition and data analysis for ankle dorsal- plantar-flexion

Two markers, distal and proximal, were placed on the tibia and a third one was placed on the top of the toe (Fig. 1, panel a). Ankle angle was approximated with the angle α defined by the line passing through the two markers placed on the tibia and the line joining the marker on the toe and the projection of malleolus on the tibia-line. The values are shifted considering 0° as the perpendicular condition. In order to reconstruct the ankle angle, first of all, a fifth-order Butterworth low-pass filter (cutoff frequency = 5 Hz) smoothed the recorded trajectories and data were projected on the plane that carried most information about the movement, identified with Principal Components Analysis [21]. For each acquisition the Mean Amplitude (MA) and the frequency (f) of the dorsal- plantar-flexion movement were calculated during active epochs. The angular displacement for the foot not performing the task during activation epochs was estimated by the Standard Deviation of α (SD) in order to verify the correct fulfillment of the task. To assess if the involuntary movement was a mirror movement or not, the correlation (R) between the angles at the two ankles was computed. Relying on values found for the healthy subject, movements of the foot which was required to stand still were considered significant when SD > 4°, which means > 5% of the moving ankle range of motion, and were considered mirror movements when R > 0.5. The training outside the bore, besides the verification of possible isometric contractions, was used even to validate the chosen landmarks as representative of the movement protocol.

### fMRI data analysis

Functional images were converted from DICOM to Analyze format with the MRIcro software [22]. Pre-processing and statistical analysis were carried out with SPM5^® ^(Wellcome Trust Centre for Neuroimaging, London, UK, http://www.fil.ion.ucl.ac.uk/spm/) running on Matlab^® ^(2007a, The MathWorks, Natick, MA).

Images were corrected for slice timing and realigned to the first image of each respective acquisition. The first acquired image is reliable because it is the first one afterward a 30 s "preparation phase", aiming at getting a steady-state magnetization. The motion correction algorithm, as a standard processing step from SPM5, was run [23].

As demonstrated by Johnstone and colleagues [24], in a block design, or more generally a design in which head motion parameters are even moderately correlated (correlation coefficient 0.2 or greater) with the model, including the head motion parameters as covariates of no interest has a deleterious impact reducing the sensitivity for detecting true activations. However, this approach, employed in several papers [e.g. 25], needs a strict inspection of the estimated realignment parameters, assessing for excessive motion.

Since our experimental design and the not negligible correlation of head motion with the required movement protocol, we chose to not insert the realignment parameters as covariates in the design matrix. In Table 1, the maximum absolute values of translation and rotation parameters for each entire session are reported; these maximum values, as expected, correspond to the last volumes of the considered session. The worst case concerns the rotational parameters for patient pre-rehabilitation acquisition performed with the left side (paretic one); she could not realize any movement and her efforts could be the main reason of these higher movement artifacts. Since this session was not used for cortical maps comparisons because of absence of any performed movement, all the others absolute values of translation indexes were less than 1.89 mm (maximum around z-axis) and rotation angles less than 2°(maximum for the pitch angle). Even if an acceptance threshold is not officially defined, these values are plentifully under thresholds already reported in literature, e.g. 4 mm translation and 5° rotation [24].

**Table 1:** Realignment parameters

		Translation (mm)			Rotation (rad)		
Subject	Session	x	y	z	Pitch	Roll	Yaw
							
Healthy	right	0.3417	0.3348	1.8892	0.0182	0.0065	0.001
	left	0.2733	0.418	1.5832	0.0165	0.0063	0.0061
Patient	Pre-right	0.8953	0.4925	0.8524	0.0234	0.0123	0.0269
	Pre-left	1.8179	1.5353	1.8285	0.0327	0.0222	0.0939
	Post-right	1.0054	0.3014	0.7574	0.0043	0.0197	0.0094
	Post-left	0.737	0.9508	1.0428	0.026	0.0171	0.0164

Maximum absolute values of translation and rotation parameters, within the realignment spatial process; they are reported for the analyzed participants, for each performed session.

Images were then normalized on the Montreal Neurological Institute (MNI) standard brain [26]. Finally, they were spatially smoothed with a Gaussian kernel homogeneous in the three spatial directions, with a Full Width Half Maximum Gaussian filter of 6 mm, to increase the signal-to-noise ratio.

For each experimental session, a general linear model was employed, performing each analysis with two different types of model design. In the first design, i.e. the standard block design, only the stimuli was modeled with a conventional boxcar function as five rest periods of 30 s alternating with five active periods of 30 s. In the second one, a user defined kinematic regressor describing the actually executed movement was added into the design matrix besides the stimuli. The kinematic regressor was the amplitude along time, computed from recorded kinematic coordinates. This way kinematic regressor comprises both different amplitude of tasks execution as well as timing of task execution not coherent with the request.

The effect of inserting the actual kinematics parameters in the generation of cortical activation maps was evaluated comparing the two models.

A high-pass filter was automatically included in the analysis by SPM5 (cutoff time constant = 128 s). Statistical analysis was accomplished using a p-value < 0.01 with Family Wise Error correction and extent threshold of 100 voxels.

Four ROIs were defined, two of them matching the representation of ankle in the sensorimotor cortex for each hemisphere and two matching the hand mapping areas. Coordinates in MNI reference system for the center (for the foot: × = ± 6 mm, y = -37 mm, z = 70 mm; for the hand: × = ± 36 mm, y = -22 mm, z = 58 mm) and extension of the ROIs were obtained from literature [27]. To define such ROIs, we used the standard software WFU PickAltas, which provides a tool for generating ROI masks based on the Talairach Daemon database; this method is an automated coordinate-based system which retrieves brain labels from the 1988 Talaraich Atlas [28].

For each acquisition, the center of mass of activated areas was calculated, weighting the intensity, of each cluster of voxels included into the areas of interest (motor ROIs).

To estimate inter-hemispheric balance, weighted laterality index (wLI) [29] was calculated from the sum of t-values across all active voxels in each ROI according to the formula:

$$\text{wLI}=\frac{\text{(}\sum t_{C}-{\sum t}_{\text{I}}\text{)}}{\text{(}\sum t_{C}+{\sum t}_{\text{I}}\text{)}}$$

where t_C _are t-values of voxels lying in the ROI in the contralateral hemisphere and t_I _are t-values of voxels lying in the ROI in the ipsilateral hemisphere. wLI ranges from -1, which stands for a totally ipsilateral activation, to 1, totally contralateral.

## Results

### Compatibility test

**Figure 2:**
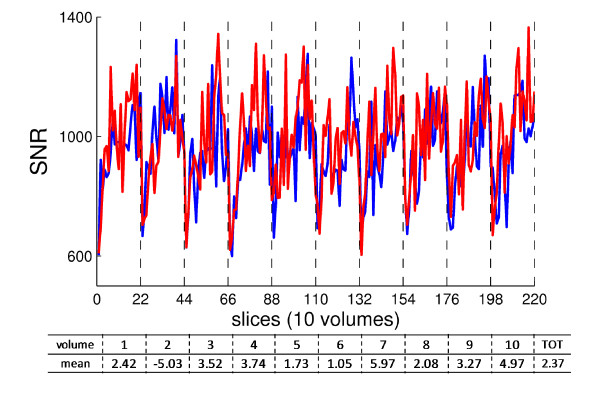
**SNR evaluation**. From gradient EPI functional acquisition on phantom, SNR along with the 220 slices, split up into 10 volumes (vertical dashed lines). Red: reference condition; Blue: with motion capture system working within the scanner room. Under the plot: table with mean of ΔSNR for each volume, and the total mean one.

The computed SNR values were compared between the two experimental conditions: reference one and with three working cameras of the motion capture system within the scanner room. In Fig 2, it is evident that the SNR was not compromised: the time profile inside one volume (22 slices) and along the acquired 30 s was the same with and without motion system, further showing an analogous reduced SNR at the first slices for each volume. In the table under the figure, the ΔSNR, within each volume, averaged on slices, and the "total" mean ΔSNR are reported, with the relative standard deviations. The relative ΔSNR, averaged among volumes, was 2.37 ± 2.9%.

Concerning the kinematic data reliability, the acceleration value, averaged among four trials, was 9.92 ± 0.26 m/s^2^, as expected in standard condition.

### Healthy subject acquisition

Healthy subject anatomical and functional images showed a similar increase in broadband noise.

On the reference anatomical images, we could see narrow zippers artifacts. As explained by Heiland in [30] they are caused by RF signals leaking into the receiver of the MR scanner and appear as bright lines in MR images. Their positions within the image depend on the frequency of the RF source that causes the artifact (not-completely shielded equipment inside the scanner room), as well as on readout bandwidth and field of view. Within the functional images, these zippers are not visible. This probably means that in functional images, the low resolution leaded to the RF noise aliasing. A basic evaluation of this the RF noise distributed on the fMRI image is represented by the SNR reduction on the phantom images.

#### 1) *Finger tapping*

Concerning the right finger tapping task, subject correctly respected the temporal sequence and performed the task with almost constant movement extent and rhythm. The entire finger tapping cycle was performed on average 11 times per activation period (0.36 Hz). No significant movement could be seen for the resting hand; indeed, ISD and PSD were both < 1% of moving index and pinkie displacements, respectively (index 0.28%; pinkie 0.83%). The correlation values (R indexes and R pinkies) were, therefore, not significant (Table 2). As expected since the accomplishment of the required protocol, the analysis with the design matrix including the kinematic regressor (index displacement along time) yielded analogous activation maps compared with the standard design matrix analysis, in terms of both localization and extensions. Activated voxels were mainly located in the sensorimotor cortex and pre-motor cortex, a few lied in Brodmann's Areas (BA) 5 and 7 too. Activation was totally contralateral (wLI = 1) and the activation barycentre was at [-37 -27 52] mm, consistent with the homunculus topography for hand. Left side provided analogous results.

**Table 2:** Kinematics of finger tapping for healthy subject

	1°PERIOD t(s): 30-60	2°PERIOD t(s): 90-120	3°PERIOD t(s): 150-180	4°PERIOD t(s): 210-240	5°PERIOD t(s): 270-300	MEAN
**ID (cm)**	3.5 ± 2	6.3 ± 2.8	8.2 ± 2.5	6.2 ± 1.6	3.8 ± 1.6	5.6 ± 2.1
**PD (cm)**	1.7 ± 0.9	3.3 ± 0.8	3.9 ± 0.9	3.1 ± 0.8	2.8 ± 0.4	3 ± 0.7
**f (Hz)**	0.33	0.37	0.33	0.37	0.4	0.36 ± 0.03
**ISD (cm)**	0.02	0.03	0.02	0.01	0.01	0.016 ± 0.007
**PSD (cm)**	0.04	0.01	0.02	0.03	0.02	0.025 ± 0.012

Kinematics data measured when the healthy subject was performing the finger tapping with the right hand. R coefficients are not reported because the two SDs were lower than 1% in all the periods.

ID: Index Displacement; PD: Pinkie Displacement; f: frequency; ISD: rest Index Standard Deviation; PSD: rest Pinkie Standard Deviation.

#### 2) *Ankle dorsal- plantar-flexion*

Concerning the dorsal-plantar-flexion of the ankle, Table 3.A and 3.B summarizes kinematics data for the healthy subject, right and left foot, respectively. As explained in *Methods*, the planarity of movement was verified for all the acquisitions by PCA: at least 98% of information related to trajectories lied on the plane chosen for projection. The subject correctly respected the temporal sequence of the task. Amplitude and frequency were repeatable across the different blocks. The foot not involved in the task was kept still (SD < 4°).

**Table 3:** Kinematics of ankle plantar- dorsi-flexion, for healthy subject and patient

		1°PERIOD t(s): 30-60	2°PERIOD t(s): 90-120	3°PERIOD t(s): 150-180	4°PERIOD t(s): 210-240	5°PERIOD t(s): 270-300	MEAN
**A**. Healthy subject right foot							
	**MA(°)**	37.89 ± 5.61	38.53 ± 4.8	43 ± 8.6	46.32 ± 10.32	49.15 ± 11.91	42.98 ± 8.24
**A**	**SD(°)**	0.81	0.3	0.43	0.46	0.2	0.45 ± 023
	**f(Hz)**	0.57	0.47	0.47	0.53	0.5	0.51 ± 0.04
	**R**	0.07	-0.2	0.35	-0.24	-0.33	-0.07 ± 0.28
**B**. Healthy subject left foot							
	**MA(°)**	46.28 ± 5.57	43.01 ± 8.16	42.39 ± 7.33	43.77 ± 7.35	44.25 ± 6.84	43.94 ± 7.05
**B**	**SD(°)**	0.89	0.99	0.48	0.49	0.45	0.66 ± 0.26
	**f(Hz)**	0.47	0.63	0.53	0.50	0.56	0.54 ± 0.06
	**R**	0.15	-0.05	0.08	-0.01	0.14	0.06 ± 0.09
**C**. Patient healthy foot at hospitalization							
	**MA(°)**	27.11 ± 7.70	29.88 ± 6.25	31.29 ± 5.91	31.8 ± 7.63	31.02 ± 7.38	30.23 ± 6.99
**C**	**SD(°)**	0.11	0.04	0.04	0.08	0.06	0.07 ± 0.28
	**f(Hz)**	0.4	0.43	0.43	0.53	0.43	0.45 ± 0.05
	**R**	-0.2	0.32	0.49	-0.07	0	0.11 ± 0.29
**D**. Patient healthy foot after one month							
	**MA(°)**	46.47 ± 7.17	44.41 ± 9.71	54.11 ± 18.37	59.95 ± 19.47	63.36 ± 18.82	53.69 ± 14.71
**D**	**SD(°)**	0.3	0.19	0.2	0.07	0.13	0.18 ± 0.33
	**f(Hz)**	0.8	0.9	0.87	0.93	0.9	0.88 ± 0.05
	**R**	-0.18	0.13	-0.07	-0.01	0.29	0.03 ± 0.18
**E**. Patient paretic foot after one month							
	**MA(°)**	9.91 ± 6.05	9.64 ± 4.7	9.74 ± 3.86	10.55 ± 4.1	11.06 ± 18.82	10.18 ± 4.72
**E**	**SD(°)**	7.8	7.77	4.93	5.6	5.34	6.58 ± 1.38
	**f(Hz)**	0.13	0.16	0.13	0.3	0.13	0.17 ± 0.07
	**R**	0.59	-0.05	0.75	0.16	0.18	0.33 ± 0.33

Ankle angle data (mean amplitude MA, standard deviation of the resting ankle SD, frequency of repetitions f, and correlation with the resting leg motion R), for:

A) Healthy subject right foot

B) Healthy subject left foot

C) Patient healthy foot at hospitalization

D) Patient healthy foot after one month

E) Patient paretic foot after one month.

**Figure 3:**
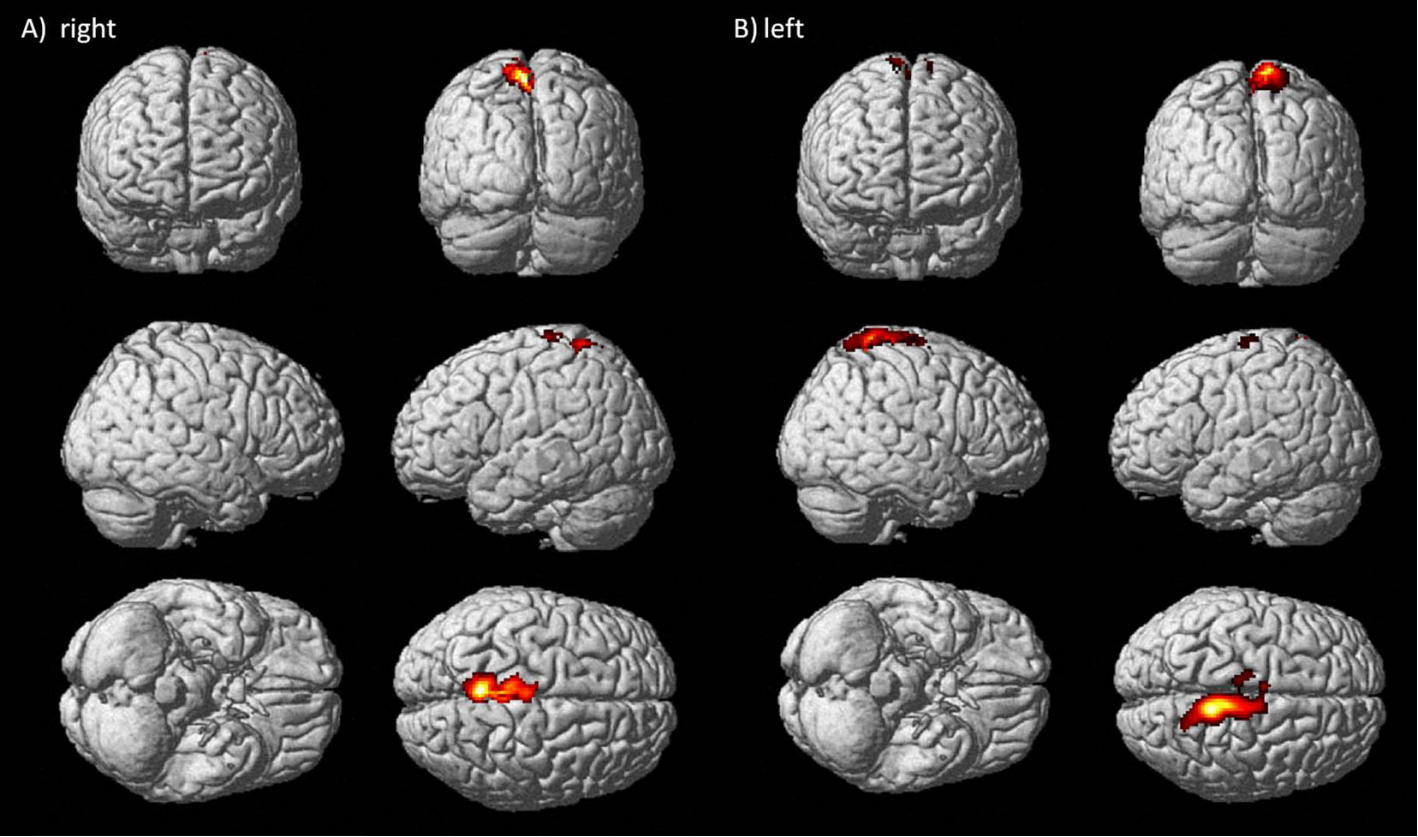
**Cortical maps for right and left ankle dorsi-flexion of healthy subject**. Activations, rendering 3D, for healthy subject, right ankle protocol (panel A) and left ankle protocol (panel B), both analyzed with the model design including the kinematic regressor.

Accordingly to the fact that the kinematic regressor (ankle angle along time) follows the pre-defined stimuli, the two analyses yielded to similar activation maps, for both sides. Activated voxels were located in controlateral sensorimotor cortex and pre-motor cortex for right ankle plantar- dorsi-flexion (Fig. 3, panel A). When executing the task with the left foot some active voxels were found in controlateral BA 5, too (Fig. 3, panel B). Activations were highly contralateral for both sides (wLI > 0.86).

For both protocols, kinematics data provided the demonstration that healthy subject performed the tasks meeting the imposed timing and using a comparable amplitude and frequency of execution along the different blocks, as expected.

### Hemiparetic subject acquisition

#### 1) Pre-rehabilitation acquisition

**Figure 4:**
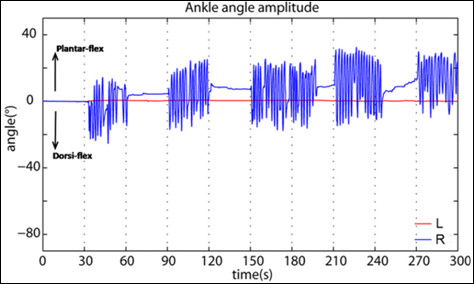
**Kinematic regressor of patient's healthy foot pre-rehabilitation**. Ankle angle amplitude of patient's healthy foot (right) at hospitalization. It was sampled for matching with the scans number and then inserted into the design matrix as kinematic regressor.

At the hospitalization the patient needed a wheelchair and could not perform any movement with the paretic limb: kinematics data did not show any significant angle variation for the paretic limb. No active voxels were found while she was trying to execute the task with the paretic foot, even when limits on cluster extension were removed and significant threshold on p-value increased till 0.05. We could hypothesize that if imagery-related activations were present, they were disorganized so as to be not visible (acute phase at hospitalization). Instead, with the healthy foot, she was able to perform the required movement, but she did not manage to meet time triggering imposed by the operator. She kept moving after stop signals in third and fourth active blocks (Fig. 4). The patient performed an average amplitude of the movement of 30.23° ± 6.99° and the frequency was 0.45 Hz ± 0.05 Hz (Table 3.C). She correctly kept still the resting leg (SD < 4°). Since she did not move one of the feet, the correlation between the two ankle angles was low (R = 0.11). In such case, given the difference between the stimuli and the kinematic performance (ankle angle along time), a modified outcome due to the kinematic regressor was expected.

**Figure 5:**
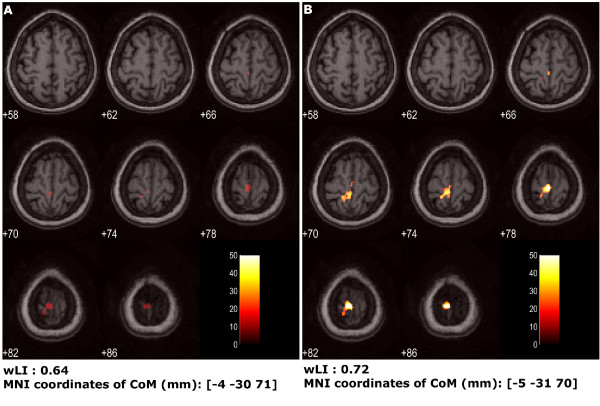
**Cortical maps of patient's healthy foot pre-rehabilitation session, comparison between the two model designs**. Activation for patient's healthy foot at hospitalization, eight transversal slices centered around z = 72 mm are shown (slice thickness = 4 mm). A) activation found using standard design matrix for statistical analysis; B) activation found using the design matrix with the kinematic regressor. Under each one, wLI and coordinates of the Center of Mass (CoM) of activated areas are reported.

Fig. 5 shows the comparison between the statistical analysis using the predefined standard block design matrix (panel A) and the matrix including the regressor with the actual kinematics (panel B). The latter led to a larger and more posterior activation (Table 4). The wLI was accordingly different (0.64 with predefined design matrix and 0.72 with kinematics regressor), being the extent of activations almost doubled. The position of activated areas barycentre was only slightly affected ([-4- 30 71] mm with predefined design matrix and [-5 -31 70] mm with kinematics regressor). Active voxels were located in the primary sensorimotor cortex and BA 5 and 7. The two involved lobes are the parietal and the frontal ones in both analyses, even if the use of kinematic regressor allows to almost duplicate the significant activated voxels in both lobes. In particular, the increased activated cortical functional BAs are within the somatosensory cortex (BA 2,3,5,7) and the motor cortex (BA 4, 6). The wider activation of BA6 indicates the strong involvement of premotor cortex (PM) and supplementary motor area (SMA).

**Table 4:** Activated voxels for not paretic pre-rehabilitation ankle plantar- dorsi-flexion session, comparing the two model designs

*Region*	*# voxels*	
	*With predefined design matrix*	*With re-defined design matrix*
**TOTAL # VOXELS**	228	451
Left cerebrum	155	336
**Parietal lobe**	103	227
Paracentral_Lobule_L (aal)	103	212
Postcentral gyrus	83	188
White matter	87	179
Gray matter	52	128
**Frontal lobe**	52	112
Precuneus_L (aal)	27	88
Paracentral lobule	35	78
Precentral gyrus	31	58
**Brodmann area 4**	16	43
**Brodmann area 3**	18	32
**Brodmann area 6**	4	26
Inter-hemispheric	11	22
Postcentral_L (aal)	14	20
**Brodmann area 5**	9	19
Medial frontal gyrus	6	15
Paracentral_Lobule_R (aal)	9	13
**Brodmann area 2**	5	6
Parietal_Sup_L (aal)	3	5
Supp_Motor_Area_R (aal)		3
Right Cerebrum		3
**Brodmann area 7**		2

Cortical and subcortical regions significantly activated during the movement protocol, obtained by the two analyses: with the predefined and with the design matrix including the kinematic regressor (FWE corrected p < 0.01).

#### 2) Post-rehabilitation acquisition

**Figure 6:**
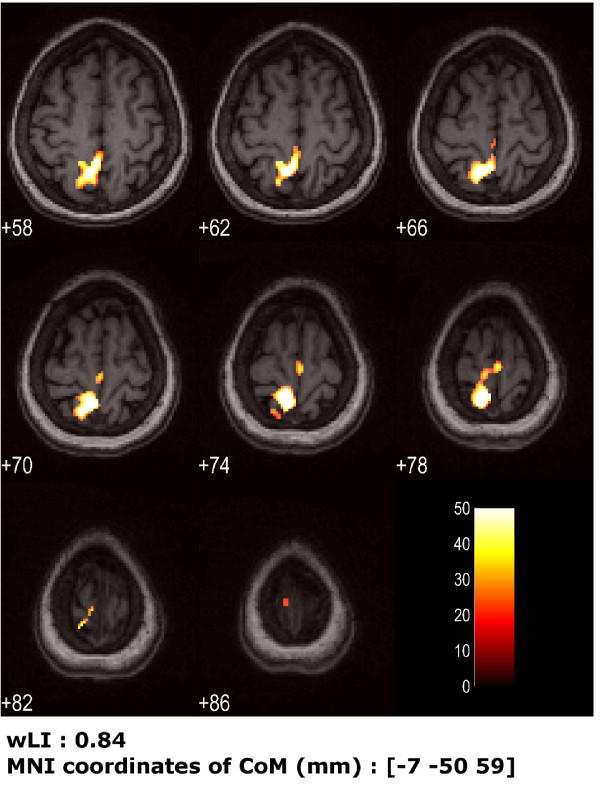
**Cortical maps for patient's healthy foot post-rehabilitation session, using the kinematics into the model design**. Activation for patient's healthy foot (right) after one month (obtained using standard design matrix), from analysis taking into account the actual kinematics. Eight transversal slices centered around z = 72 mm are shown (slice thickness = 4 mm). Under the figure, wLI and coordinates of the Center of Mass (CoM) of activated areas are reported.

After one month of rehabilitation, for the not impaired limb, the patient achieved a good fulfillment of temporal sequence; the ankle motion was quite repeatable in between blocks. As a consequence, the model design with the addition of the kinematic regressor did not modify significantly the activation maps. The amplitude of the movement was 53.69° ± 14.71° performed at a frequency of 0.88 Hz ± 0.05 Hz. The resting limb produced a SD < 4° with no correlation with the moving side (R = 0.03, Table 3.D). Primary sensorimotor cortex and BA 7 (Fig. 6), prevalently in the contralateral hemisphere (wLI = 0.84), were activated. Compared to the pre-rehabilitation session of the same foot, these findings highlighted a globally larger activated area and a slight improving of the controlaterality.

After one month of rehabilitation the patient was able to move again the paretic side. With the paretic limb the patient executed a movement of 10.18° ± 4.72° at 0.17 Hz ± 0.07 Hz (Table 3.E).

**Figure 7:**
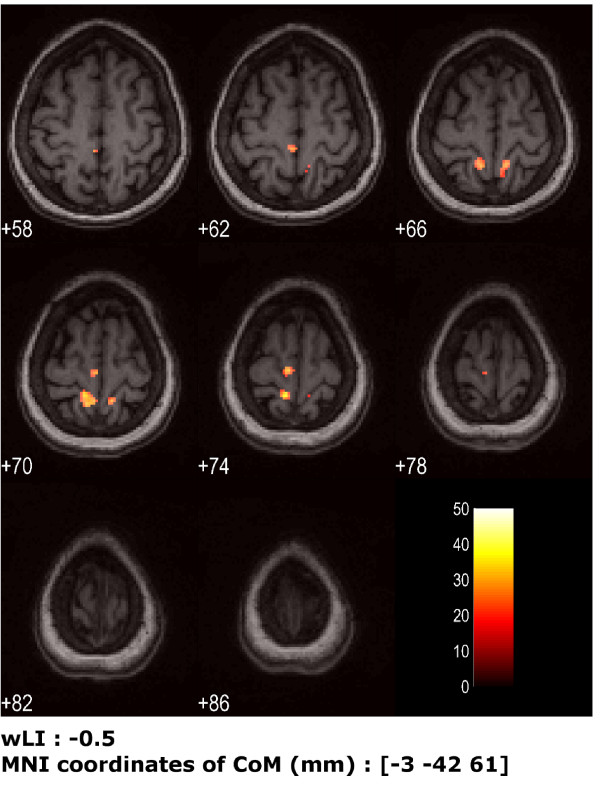
**Cortical maps for patient's paretic foot post-rehabilitation session, using the kinematics into the model design**. Activation for patient's paretic foot (left) after one month given by analysis taking into account the actual kinematics. Eight transversal slices centered around z = 72 mm are shown (slice thickness = 4 mm Under the figure, wLI and coordinates of the Center of Mass (CoM) of activated areas are reported.

Nevertheless the patient did not manage either to meet the task timing or to keep the right foot still, as requested by the protocol; the SD of the supposed resting foot was 6.58° ± 1.38°; the correlation between the feet was R = 0.33. The activations obtained from the two model designs were different. In particular, the standard design yielded to small clusters (all less than 25 voxels) and all in the ipsilater hemisphere. Instead, inserting the actual kinematics regressor into the design matrix yielded to more meaningful activation maps, i.e. wider clusters and even in the controlateral hemisphere (Fig. 7).

## Discussion

### Compatibility test

We can assess that the loss of SNR introduced by the motion system (2.37 ± 2.9%) is negligible. Indeed, we can use as reference the recent study of Scarff and colleagues [31]: in simultaneous recordings of fMRI and EEG, they showed that MR image SNR, computed as we did, decreased as the number of electrodes increased, and they fix as data quality acceptable a SNR loss on the images of 11-12%. Their value originates from completely different device components, more complex and necessarily closer to the MR scanner; anyway, it can be considered a general reference (worst case) about the additive noise on the fMRI images due to a new device. Other studies [32,33] using similar parameters (e.g. signal to noise fluctuation ratio) assessed the reliability of fMRI images finding a relative SNR loss with respect to the standard condition of 2.75%. Mullinger and colleagues [34] evaluated on a phantom the effect of the conducting materials in the EEG-caps with 1.5 T acquisitions, accepting a SNR reduction of 27% with 32 electrodes.

The use of three cameras allows a reliable reconstruction of 3D positions of the markers. Three cameras, even if not positioned with the optimal mutual orientations having as major priority to put them on the ceiling at the maximal distance from the bore, represent a good compromise between the introduced noise and the reliability of markers reconstruction. Indeed, the calibration procedure for each session, estimating the reconstruction error on a moving bar with 3 markers at fixed known distances, confirms the high accuracy of kinematics data (in our case, accepted error < 1 mm on a working volume of 1×1×1 m).

The computed mean gravity acceleration was as the expected one, hence the magnetic fields did not affect the motion capture system and camera data processing.

Feasibility of methodology was therefore demonstrated.

### Healthy subject acquisition

Both the hands and the legs were visible; thus, excluding the part inserted into the bore, it was demonstrated the possibility of acquiring a great number of multi-segment motor tasks. Since the easiness and the not invasiveness of markers positioning, the landmarks definition can be customized depending on the patient's specific movement skills and the segments involvement in the movement execution. For instance, depending on the goals of study, it could be necessary, for a finger tapping task, to monitor each individual finger. Smaller markers and not cumbersome rigid structures could represent valid solutions, but the working volume extent, the distance between cameras and bore and the reconstruction error have to be specifically evaluated.

On the healthy subject anatomical image two narrowband "zippers" appeared. Because of their position and size, no problem occurred for image processing. However, loss of significance could not be completely excluded due to pixels covered by zippers.

For both the tested protocols on the healthy subject, we implemented the proposed method including the actual kinematics into the protocol model. As expected for healthy subject, who correctly meet the request, the kinematic parameter did not add new information with respect to the pre-defined stimuli and the cortical maps did not experience any significant changes.

The activation maps areas, the position of clusters barycentres and the level of controlaterality were in both the tests consistent with the literature. Comparing the obtained functional areas between the two motor tasks, it was highlighted an additional activation of BA 5 and 7 for finger tapping compared to ankle dorsal- plantar-flexion. Indeed, these areas are involved in maintaining a spatial reference system during execution of fine and complex tasks, by coordinating movement and proprioception, hence when the involved degrees of freedom are numerous. The hand has a larger cortical representation, especially in the pre- and postcentral gyri, compared to lower limb representations [7-35], as expected by literature.

### Hemiparetic subject acquisition

The healthy foot pre-rehabilitation and the paretic foot post-rehabilitation sessions confirmed the usefulness of design matrix redefinition with the inclusion of the kinematic data. In the latter, only with such model optimization activation maps showed significant activation clusters, making the cortical map consistent with the performed bilateral modest movements.

Furthermore, for healthy foot post-rehabilitation session, we obtained a greater extension of activations, in the same BAs, compared to the ones found at hospitalization, before the rehabilitation treatment. We have to consider that the observed difference in the activation areas could be linked to the greater amplitude of the movement (30.23° ± 6.99° pre, 53.69° ± 14.71° post) and the higher frequency of execution (0.45 Hz ± 0.05 Hz pre, 0.88 Hz ± 0.05 Hz post). The quantitative measurements of movement amplitude and frequency obtained with motion capture system provide information that could be precious to relate difference in activation characteristics to difference in the movement parameters. A recent study [36] evidenced that post-stroke modifications in neuronal networks controlling the paretic limb, especially compensatory recruitment of the non-lesioned hemisphere, may affect cortical areas in control of the non-paretic limb. Moreover, non-use of both lower extremities due to impaired walking or altered limb kinematics and body posture due to hemiparesis may induce neural adaptations in networks controlling the intact limb. Hence, quantifying mirror movements and movement extent, both for paretic and healthy sides, is crucial to interpret what is due to bilateral movements, what is due to larger movements and what is an expression of neural plasticity: indeed, depending on lesion location, a compensatory recruitment of bilateral cortical regions can be part of the motor recovery.

The standard statistical analysis of fMRI images, usually employed in clinical examinations, is based on the repeatability of protocol blocks, in terms of both periods duration and execution parameters (amplitude and frequency). This hypothesis is actually the main limitation of fMRI exploitation for motor recovery evaluation; indeed, this repeatability is not quantitatively verified, thus the resulting cortical maps are affected by possible variations of the task execution. This repeatability assumption becomes even weaker for neurological patients than for healthy subjects. The possible poor matching among protocol blocks parameters can affect the intra-session analysis. This non-repeatability increases when considering different sessions of the patient at different stages of the rehabilitative pathway; this element needs therefore to be monitored for longitudinal studies aimed at the evaluation of rehabilitative process. This loss of comparability turns out to be even more significant for inter-subjects studies, where, for instance, a specific rehabilitation treatment is under test.

The repeatability of the markers placement and the comparability of motion parameters represent the main advantage of using motion capture system with respect to EMG, where the level of noise of the recorded signal and the criticality of electrodes positions strongly limit the possibility to compare consistently muscles activation profiles between different experimental sessions. Further, when the interest is on movement execution, the correct single subject choice of muscles to be studied can complicate because of synergism, while kinematics offer a simple, reliable and general picture of motion. On the contrary, when the study is focused on presence of isometric contractions, kinematics is not at all suitable, or when different muscles strategies are investigated only EMG could provide detailed analysis.

The present work demonstrates the availability of the possible simultaneous measure of kinematics data and fMRI, offering an innovative and extremely flexible experimental set-up for a better understanding of neural correlates of motor tasks.

As initial step, here the kinematics data have been successfully adopted to enrich the design matrix by including the representative parameters of the performed movement during fMRI block statistical processing; it means to take into account both the movement extent within and between blocks and the actual specific segmentation of task-execution periods and rest periods. The utility of design matrix re-definition for fMRI statistical processing have been recently demonstrated also by Krainak and colleagues [11]: the mechanical motor output was measured in terms of force and torque, by a MR compatible 6 degree of freedom load cell, and the torque signal was used to identify the onset and the end of each single trial; the set-up permitted nevertheless only isometric protocols for upper limbs. Our combined methodology allows, indeed, recording of multi-joint dynamic motor tasks and there are not any constraints about the duration of trials, which can be defined for both block or event-related protocols.

Moreover, the use of motion capture allows to track a great number of markers in the calibrated working volume, permitting synchronized quantitative information about movements of multiple segments. This aspect strongly impacts on mirror movements monitoring, which allows to correctly interpret possible ipsilateral activations, distinguishing between activations due to movements of the limb which was asked to be still and activations due to a cortical reorganization as form of motor recovery. The accuracy of the motion system allows to detect even mirror movements with amplitude smaller than 0.5 cm, i.e. angles about < 2°; therefore also not visible movements, almost flickers, are turned out by the system. Recently, Enzinger and colleagues [6] carried out an fMRI ankle dorsiflexion paradigm to test for cortical reorganization in patients with chronic stroke with varying degrees of residual gait impairment. A wooden ankle support with an electrogoniometer was used. Since the most interesting results concern the increased cortical activation in the unlesioned hemisphere (ipsilateral to paretic limb), it could be very enriching to apply a complete kinematic analysis, able to provide a quantification of probable mirror movements and a global 3-dimensional multi-segment measurement of lower limbs.

Another challenging application of fMRI simultaneous kinematic analysis could be in the investigations of functional properties of brain areas associated with motor execution and imagery [37], with the final goal to understand the effectiveness of motor imagery to enhance the recovery. The kinematics recordings could provide a method solving the main issue concerning the feedback of motor imagery task accuracy; indeed, it could verify the absence of any actual movement, even if isometric contractions not resulting in motion could be masked by kinematic acquisitions.

In order to systematically verify correlations between motor output and cortical activations, more complex protocols should be employed, with more detailed instructions to the subject: established frequencies and amplitudes should be kept constant for defined sessions or systematically changed as request. Such complexity could be unfeasible for many neurologic patients and a quantitative instrumentation for objective movement monitoring is needed, able to detect even undesired or unconscious variations in the motor task.

A complete and structured analysis of the effects of motor execution parameters to the activation maps in healthy and in pathological subjects will be necessary to provide reliable information for the clinical massive use of motor fMRI acquisitions. Whether and in which extent there could be a relation between kinematics parameters and activation area will be the object of following deeper experimental studies. In literature the amplitude effect was studied, e.g. with a simple finger tapping test [38], supporting the hypothesis that a larger amplitude of the task would correspond to a larger BOLD signal. Similar suggestions came from MachIntosh's studies on ankle dorsiflexion, measured by fiberoptic device on one joint: large-amplitude movements yielded to less lateralized activation compared to small-amplitude movements, after verification of no difference in relative head motions [7]. Multi-segment and bilateral kinematics monitoring could add useful information to these hypotheses. Indeed, as far as we know, no generalization and systematic findings about the amplitude role on cortical activations are shown. Frequency parameter on movement execution is more popular in literature even if opposing results were asserted. Some studies did not find any relationship between frequency and activation areas [39], on the contrary others [40] demonstrated the parallel increasing of movement frequency and BOLD signal; finally, Sadato et al [41] showed the size of activated area increased with higher frequencies only up to 2 Hz. There is still great uncertainty concerning these relationships, analyzing different motor tasks.

Our proposed combined recording of motor output and neural correlates performs a continuous movement monitoring, including different time-varying kinematics parameters as regressors in the fMRI processing, so optimizing the protocol model with the movement output [42]. This methodology should provide a more precise reduction in the number of uncontrolled variables, enhancing the capability to discern the causes of different cerebral activations: motor performance characteristics or cortical reorganization.

## Conclusions

As a general conclusion, with respect to the current gold standard for motor output assessment during fMRI, i.e. MR-compatible EMG acquisition, we highlight some advantages which could promote the use of motion capture system to enrich EMG data or to substitute EMG, depending on the research goals.

Firstly, since the kinematics is well known to be much reliable in terms of markers positioning, both intra-subject and inter-subjects, the motion analysis during fMRI can be well applicable to different subjects and to different experimental conditions, allowing solid comparisons. EMG data are difficult to be repeatable even on the same subject, as extremely affected by electrodes placement. Moreover, significantly different muscular synergies could be adopted by subjects, leading to the need of detecting many muscles to get a complete information about performed movement. Secondly, kinematics allows multi-segments acquisitions, providing a bilateral and complete description of motor task execution, through quantified parameters such as start and end instants of movement, amplitude, frequency, and verification of mirror movements.

On the other hand, there are some technical disadvantages for kinematics versus EMG. The first is the lost of isometric contractions; to overcome this issue it is possible or to verify before the fMRI protocol the existence of isometric contractions (as in this work), or to couple EMG and kinematics, exploiting the strength points of each methodology, during the fMRI examination. Similarly, when muscles synergies are under investigation only EMG is feasible. A further weak issue concerning kinematics is that the scientific community in neuroimage is now acquainted to EMG, and the comparison between EMG studies and kinematics parameters is not immediate and requires some preliminary investigations.

## Competing interests

The authors declare that they have no competing interests.

## Authors' contributions

CC participated to study design, data collection and analysis, and manuscript writing; SF participated to study design, data collection and analysis, and manuscript definition; MG participated to data analysis and methods definition, to literature comparisons and manuscript revisions; NV participated in literature overview, in the data collection and in the preliminary analysis; GF participated to study design and compatibility assessment; GB participated to fMRI images processing and statistical analysis; TF participated to data collection and neurophysiological interpretation; AM participated to study design and to clinical assessment; FM participated to recruitment of stroke patients and rehabilitation treatment evaluation; AP participated to study design, data collection and analysis and manuscript revision.

All authors read and approved the final manuscript.
